# Supplementary material for: Spontaneous pauses in firing of external pallidum neurons are associated with exploratory behavior
Source: Commun Biol. 2022 Jun 21;5:612. doi: 10.1038/s42003-022-03553-z (PMC9213498; doi:10.1038/s42003-022-03553-z)
Supplement: Supplementary file 2 — Supplementary Information [file 42003_2022_3553_MOESM2_ESM.pdf]

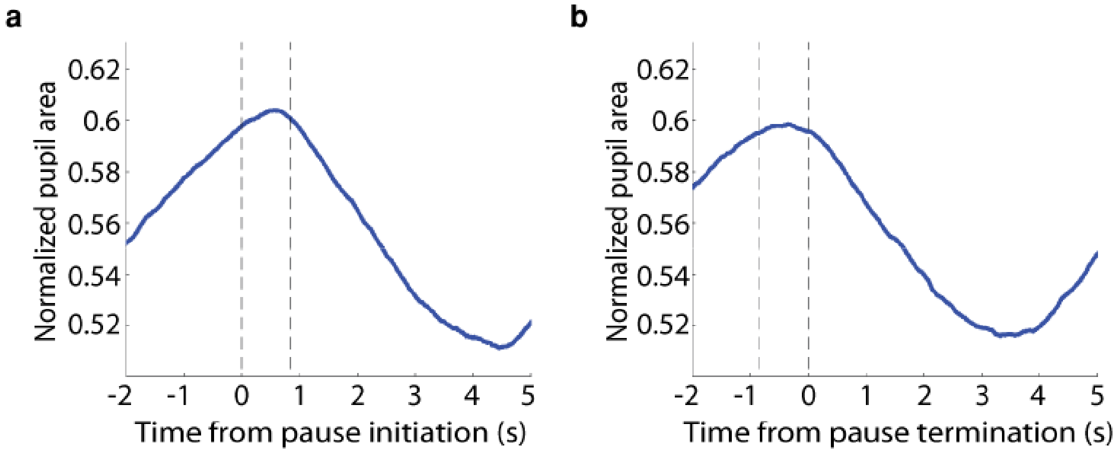

Supplementary Figure 1. Pupils dilate around GPe pauses.

(a) Average pupil area around pauses (*n* = 31,247 pauses). Abscissa, time (−2 to 5 s); ordinate, pupil area (scaled using min-max normalization). The vertical dashed lines at t = 0 and t = ~0.85 s indicate the time of pause initiation and mean pause termination, respectively. Only pauses that were recorded simultaneously with the pupil area data were included in the analysis. **(b)** Same as (a), but the vertical dashed lines at t = 0 and t = ~-0.85 s indicate the time of pause termination and mean pause initiation, respectively.

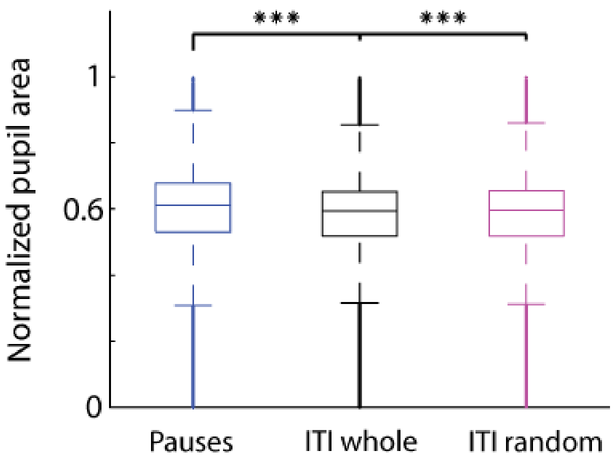

Supplementary Figure 2. Pupil area is on average higher during pauses than in ITIs with no pauses.

Boxplot showing the mean pupil areas during pauses (blue; n = 31,247 pauses), mean pupil areas in ITIs with no pauses, computed for the entire last 4 s of the ITI (black; n = 63,572 ITIs) and mean pupil areas in randomly selected 0.85-s periods from the last 4 s of the ITIs with no pauses (magenta; n = 63,572 ITIs). The random periods (N = 100) were drawn from a uniform distribution, and the corresponding pupil area values were averaged. Center line of the boxplot indicates the median value, and bottom and top hinges indicate the first and third quartiles of data, respectively. The whiskers extend from the bottom and top hinges to a distance ≤1.5× interquartile range. Outliers extend beyond the whiskers and are plotted individually. ***$P < 0.001$, two-sided Mann–Whitney U test.

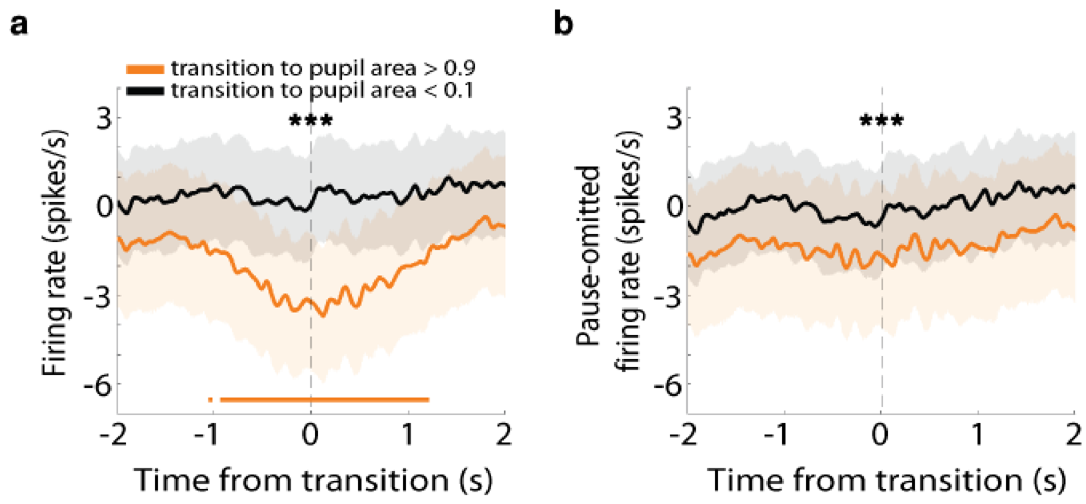

Supplementary Figure 3. The normalized firing rate by itself was not strongly associated with changes in pupil area.

(a) Average population dynamics of the discharge rate for different normalized pupil areas. Abscissa, time (−2 to 2 s), zero is the time when the normalized pupil area crossed to values < 0.1 (black curve; n = 16,995 transitions) and to values > 0.9 (orange curve; n = 9,556 transitions); ordinate, firing rate in Hz, normalized by the mean discharge rate during the last 4 s of the ITI period. The orange curve is significantly lower than the black curve (***$P < 0.001$, two-sided Mann–Whitney U test). The horizontal lines indicate activity lower than the mean minus three standard deviations of the firing rate on the interval from -2 s to -1.5 s. **(b)** Average population dynamics of the discharge rate after removal of pause-containing segments. Same conventions as in (a).

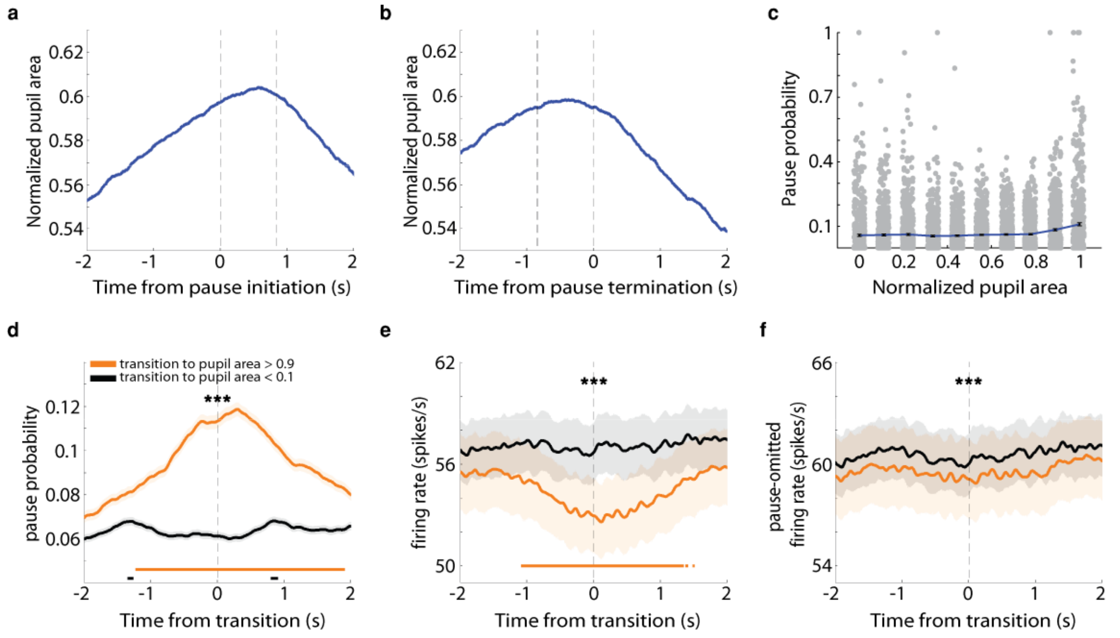

Supplementary Figure 4. GPe pause probability is increased when the pupils are dilated, even when considering only periods of time when no saccades were made.

(a) Average pupil area around pauses (*n* = 31,247 pauses). Abscissa, time (−2 to 2 s); ordinate, pupil area (scaled using min-max normalization). The vertical dashed lines at t = 0 and t = ~0.85 s indicate the time of pause initiation and mean pause termination, respectively. Shaded regions represent s.e.m. Only pupil area data from time periods when no saccades were made were considered for analysis (pupil area values at the time of saccades were replaced with NaNs). **(b)** Same as (a), but the vertical dashed lines at t = 0 and t = ~-0.85 s indicate the time of pause termination and mean pause initiation, respectively. **(c)** Average population pause probability as a function of normalized pupil area. For each neuron and for each range of pupil area values (0-0.1, 0.1-0.2,…, 0.8-0.9, 0.9-1), the total time the neuron paused was divided by the overall time the pupil was in a particular area range. Each dot represents data from a single neuron (*n* = 579 neurons). Error bars represent s.e.m. Only data from time periods when no saccades were made were considered for analysis. **(d)** Average population dynamics of pause probability for different normalized pupil areas. Abscissa, time (−2 to 2 s), zero is the time when the normalized pupil area crossed to values < 0.1 (black curve; *n* = 17,507 transitions) and to values > 0.9 (orange curve; *n* = 10,605 transitions); ordinate, pause probability. The orange curve is significantly higher than the black curve (****P* < 0.001, two-sided Mann–Whitney *U* test). The horizontal lines indicate activity greater than the mean plus three standard deviations of the pause probability on the interval from -2 s to -1.5 s. The pause probability was computed using 1-ms bins and smoothed with a Gaussian window with a s.d. of 20 ms. Shaded regions represent s.e.m. Only data from time periods when no saccades were made were considered for analysis. **(e)** Average population dynamics of discharge rate for different normalized pupil areas. Abscissa, time (−2 to 2 s), zero is the time when the normalized pupil area crossed to values < 0.1 (black curve; *n* = 17,507 transitions) and to values > 0.9 (orange curve; *n* = 10,605 transitions); ordinate, firing rate in Hz. The orange curve is significantly lower than the black curve (****P* < 0.001, two-sided Mann–Whitney *U* test). The horizontal lines indicate activity lower than the mean minus three standard deviations of the firing rate on the interval from -2 s to -1.5 s. **(f)** Average population dynamics of discharge rate after removal of pause-containing segments. Same conventions as in (e).

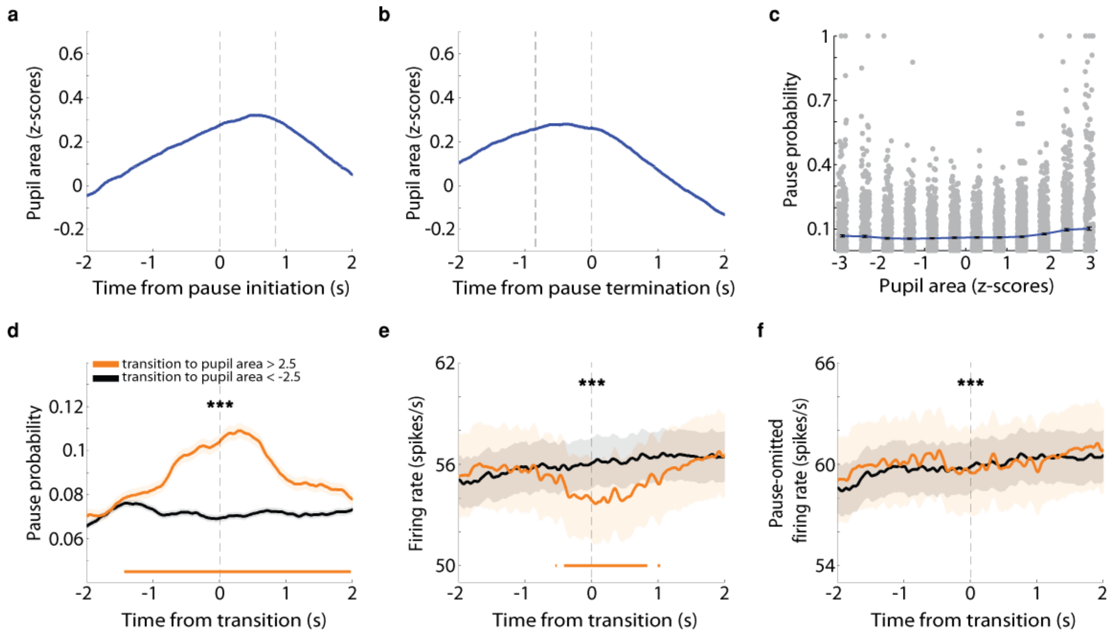

Supplementary Figure 5. GPe pause probability is increased when the pupils are dilated, regardless of the normalization method used.

(a) Average pupil area around pauses (*n* = 31,247 pauses). Abscissa, time (−2 to 2 s); ordinate, pupil area (normalized using z-scores). The vertical dashed lines at t = 0 and t = ~0.85 s indicate the time of pause initiation and mean pause termination, respectively. Shaded regions represent s.e.m. **(b)** Same as (a), but the vertical dashed lines at t = 0 and t = ~-0.85 s indicate the time of pause termination and mean pause initiation, respectively. **(c)** Average population pause probability as a function of *z*-score-normalized pupil area. For each neuron and for each range of pupil area values (-3 to -2.5, -2.5 to -2, -2 to -1.5,…, 2 to 2.5, 2.5 to 3), the total time the neuron paused was divided by the overall time the pupil was in a particular area range. Each dot represents data from a single neuron (*n* = 579 neurons). Error bars represent s.e.m. **(d)** Average population dynamics of pause probability for different normalized pupil areas. Abscissa, time (−2 to 2 s), zero is the time when the normalized pupil area crossed to *z*-score < -2.5 (black curve; *n* = 23,971 transitions) and to *z*-score > 2.5 (orange curve; *n* = 8,735 transitions); ordinate, pause probability. The orange curve is significantly higher than the black curve (***$P < 0.001$, two-sided Mann–Whitney *U* test). The horizontal line indicates activity greater than the mean plus three standard deviations of the pause probability on the interval from -2 s to -1.5 s. The pause probability was computed using 1-ms bins and smoothed with a Gaussian window with a s.d. of 20 ms. Shaded regions represent s.e.m. **(e)** Average population dynamics of discharge rate for different normalized pupil areas. Abscissa, time (−2 to 2 s), zero is the time when the normalized pupil area crossed to *z*-score < -2.5 (black curve; *n* = 23,971 transitions) and to *z*-score > 2.5 (orange curve; *n* = 8,735 transitions); ordinate, firing rate in Hz. The orange and black curves come from different distributions (***$P < 0.001$, two-sided Mann–Whitney *U* test). The horizontal lines indicate activity lower than the mean minus three standard deviations of the firing rate on the interval from -2 s to -1.5 s. **(f)** Average population dynamics of discharge rate after removal of pause-containing segments. Same conventions as in (e).

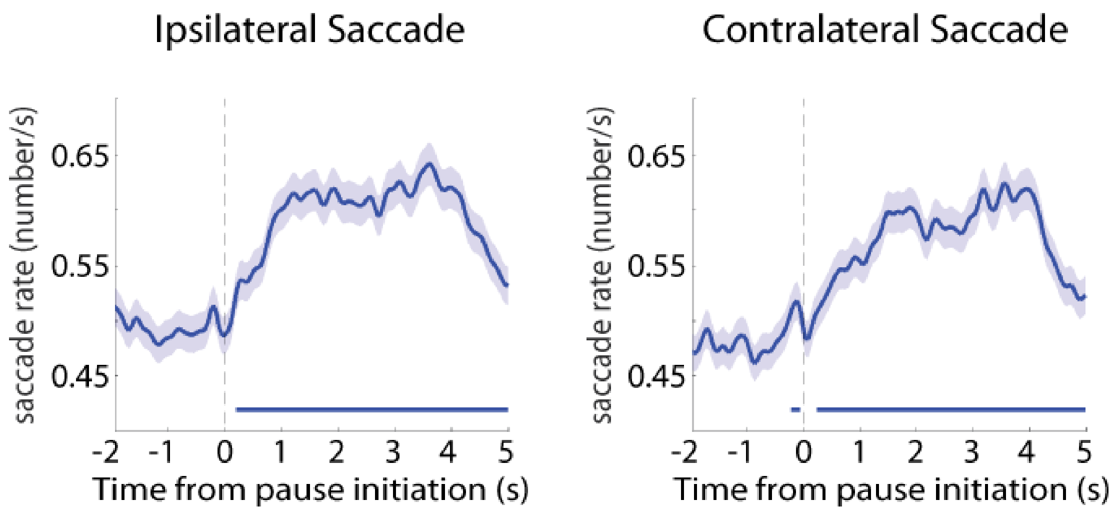

Supplementary Figure 6. Saccades to the contralateral visual field are not more abundant around GPe pauses than saccades to the ipsilateral visual field.

Average saccade rate to the ipsilateral and contralateral visual fields around pauses (n = 33,798 pauses). Abscissa, time (−2 to 5 s, time zero indicates pause initiation); ordinate, saccade rate in Hz. The horizontal lines indicate activity greater than the mean plus three standard deviations of the saccade rate on the interval from -2 s to -1.5 s. Saccade rate was computed using 50-ms bins and smoothed with a Gaussian window with a s.d. of 1 ms. Shaded regions represent s.e.m.

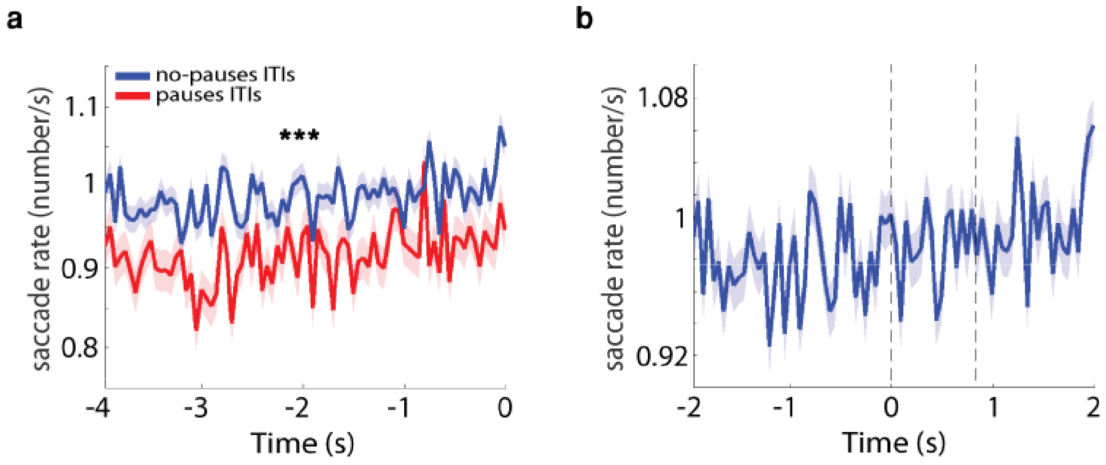

Supplementary Figure 7. The saccade rate is on average higher in ITIs without pauses than in ITIs with at least one pause.

(a) Timeline of the average saccade rate in the last 4 s of the ITIs with no pauses (blue, "no-pauses ITIs"; *n* = 68,304 ITIs) and ITIs with at least one pause (red, "pauses ITIs"; *n* = 25,547 ITIs). Abscissa, time (−4 to 0 s), zero is end of the ITI; ordinate, saccade rate in Hz. Saccade rate was computed using 50-ms bins. Shaded regions represent s.e.m. The saccade rates in the no-pauses ITIs are significantly higher than the saccade rates in the pauses ITIs. ***$P < 0.001$, two-sided Mann–Whitney *U* test**.** **(b)** Average saccade rate in the no-pauses ITIs (*n* = 68,304 ITIs). Abscissa, time (−2 to 2 s); ordinate, saccade rate in Hz. The vertical dashed lines at t = 0 and t = ~0.85 s indicate the times of mean pause initiation and mean pause termination (computed from the pauses ITIs), respectively. Saccade rate was computed using 50-ms bins. Shaded regions represent s.e.m.
