# Supplementary material for: Spontaneous pauses in firing of external pallidum neurons are associated with exploratory behavior
Source: Commun Biol. 2022 Jun 21;5:612. doi: 10.1038/s42003-022-03553-z (PMC9213498; doi:10.1038/s42003-022-03553-z)
Supplement: Supplementary file 3 — Description of Additional Supplementary Files [file 42003_2022_3553_MOESM3_ESM.docx]

**Description of Additional Supplementary Files**

**File name:** Supplementary Data 1
**Description:** : The source data behind the graphs presented in the main figures of the paper
