# Supplementary material for: Spontaneous pauses in firing of external pallidum neurons are associated with exploratory behavior
Source: Commun Biol. 2022 Jun 21;5:612. doi: 10.1038/s42003-022-03553-z (PMC9213498; doi:10.1038/s42003-022-03553-z)
Supplement: Supplementary file 5 — Reporting Summary [file 42003_2022_3553_MOESM5_ESM.pdf]

nature portfolio

Corresponding author(s): Alexander Kaplan

Last updated by author(s): May 8, 2022

Reporting Summary

Nature Portfolio wishes to improve the reproducibility of the work that we publish. This form provides structure for consistency and transparency in reporting. For further information on Nature Portfolio policies, see our Editorial Policies and the Editorial Policy Checklist.

Statistics

For all statistical analyses, confirm that the following items are present in the figure legend, table legend, main text, or Methods section.

n/a	Confirmed	
☐	☒	The exact sample size (*n*) for each experimental group/condition, given as a discrete number and unit of measurement
☐	☒	A statement on whether measurements were taken from distinct samples or whether the same sample was measured repeatedly
☐	☒	The statistical test(s) used AND whether they are one- or two-sided<br>*Only common tests should be described solely by name; describe more complex techniques in the Methods section.*
☐	☒	A description of all covariates tested
☒	☐	A description of any assumptions or corrections, such as tests of normality and adjustment for multiple comparisons
☐	☒	A full description of the statistical parameters including central tendency (e.g. means) or other basic estimates (e.g. regression coefficient) AND variation (e.g. standard deviation) or associated estimates of uncertainty (e.g. confidence intervals)
☐	☒	For null hypothesis testing, the test statistic (e.g. *F*, *t*, *r*) with confidence intervals, effect sizes, degrees of freedom and *P* value noted<br>*Give P values as exact values whenever suitable.*
☒	☐	For Bayesian analysis, information on the choice of priors and Markov chain Monte Carlo settings
☒	☐	For hierarchical and complex designs, identification of the appropriate level for tests and full reporting of outcomes
☒	☐	Estimates of effect sizes (e.g. Cohen's *d*, Pearson's *r*), indicating how they were calculated

Our web collection on statistics for biologists contains articles on many of the points above.

Software and code

Policy information about availability of computer code

Data collection	The spiking activity data in this study were collected using a template matching software (SnR, software version: 2.0.0; Alpha Omega Engineering). The pupil size and eye position data were collected using a data acquisition and control software (DQW; ISCAN Incorporated).
Data analysis	All data analysis were performed using custom software, written on MATLAB 2013a (Mathworks, MA, USA). The code related to this study is available from the corresponding author upon request.

For manuscripts utilizing custom algorithms or software that are central to the research but not yet described in published literature, software must be made available to editors and reviewers. We strongly encourage code deposition in a community repository (e.g. GitHub). See the Nature Portfolio guidelines for submitting code & software for further information.

Data

Policy information about availability of data

All manuscripts must include a data availability statement. This statement should provide the following information, where applicable:

- Accession codes, unique identifiers, or web links for publicly available datasets
- A description of any restrictions on data availability
- For clinical datasets or third party data, please ensure that the statement adheres to our policy

Data from this study are available from the corresponding author upon request.

March 2021

Field-specific reporting

Please select the one below that is the best fit for your research. If you are not sure, read the appropriate sections before making your selection.

☒ Life sciences ☐ Behavioural & social sciences ☐ Ecological, evolutionary & environmental sciences

For a reference copy of the document with all sections, see nature.com/documents/nr-reporting-summary-flat.pdf

Life sciences study design

All studies must disclose on these points even when the disclosure is negative.

Sample size	Our selection of sample size was based on conventions of non-human primate research studies. We therefore used two monkeys. For each monkey, we recorded the activity of a few hundreds of single-units in the external segment of the globus pallidus (GPe).
Data exclusions	Our single-unit data was selected based on predefined inclusion criteria of the isolation quality (isolation score and firing rate stability; see Methods section). Single-units that did not pass the inclusion criteria were excluded from the dataset.
Replication	To verify the reproducibility of our findings, we used two monkeys in the experiment. Our results were qualitatively similar between monkeys.
Randomization	Randomization was not relevant to our study, since we did not have different experimental groups/conditions.
Blinding	Blinding was not relevant to our study, since we did not allocate our animal subjects into different groups.

Reporting for specific materials, systems and methods

We require information from authors about some types of materials, experimental systems and methods used in many studies. Here, indicate whether each material, system or method listed is relevant to your study. If you are not sure if a list item applies to your research, read the appropriate section before selecting a response.

Materials & experimental systems

n/a	Involved in the study	
☒	☐	Antibodies
☒	☐	Eukaryotic cell lines
☒	☐	Palaeontology and archaeology
☐	☒	Animals and other organisms
☒	☐	Human research participants
☒	☐	Clinical data
☒	☐	Dual use research of concern

Methods

n/a	Involved in the study	
☒	☐	ChIP-seq
☒	☐	Flow cytometry
☒	☐	MRI-based neuroimaging

Animals and other organisms

Policy information about studies involving animals; ARRIVE guidelines recommended for reporting animal research

Laboratory animals	Female African green monkeys (Vervets, Chlorocebus aethiops sabaeus), 5-8 years old (young adults).
Wild animals	The study did not involve wild animals.
Field-collected samples	The study did not involve samples collected from the field.
Ethics oversight	The experimental protocol was approved and supervised by the Institutional Animal Care and Use Committee of the faculty of medicine, the Hebrew University, Jerusalem, Israel.

Note that full information on the approval of the study protocol must also be provided in the manuscript.

March 2021
